# Facial and body contouring with 1444 nm Nd:YAG laser‐assisted lipolysis: Clinical evidence

**DOI:** 10.1111/srt.13400

**Published:** 2023-06-28

**Authors:** Domenico Piccolo, Mohammed Hussein Mutlag, Irene Fusco, Paolo Bonan

**Affiliations:** ^1^ Skin Center – Dermo Aesthetic Laser Centers Avezzano, Pescara and L'Aquila Avezzano Italy; ^2^ Roma clinic Baghdad Iraq; ^3^ Clinical Research and Practice El.En. Group Calenzano Italy; ^4^ Laser Cutaneous Cosmetic and Plastic Surgery Unit villa Donatello Clinic Florence Italy

**Keywords:** 1444 nm Nd:YAG laser, facial and body contouring, GAIS scale, lipolysis

## Abstract

**Background:**

Body contouring is a significant area of dermatologic and plastic surgery. Surgical procedures, like surgical lipectomy, and less invasive procedures, such as various liposuction techniques, are the two main ways to reduce fat.

**Aim:**

Our study showed that 1444 nm Nd:YAG laser‐assisted lipolysis used with appropriate and specific parameters effectively destroys adipose tissue avoiding these risks and determining a safe clinical application.

**Method:**

A subcutaneous, 1444 nm Nd:YAG laser was used on 132 patients (range, 18–73 years; 109 women and 23 men with Fitzpatrick skin phototypes ranging between II and V) requiring body and face contouring. All patients were photographed for documentation. Digital clinical photography was taken under as near identical conditions as possible at baseline (pre‐treatment), and 16 weeks post‐treatment. The 5‐point Global Aesthetic Improvement Scale (GAIS) was recorded immediately after treatment and at their final assessment session (4 months). Adverse events were monitored.

**Results:**

On the basis of the investigator‐evaluated GAIS scale, the total GAIS scores showed satisfactory results. Clinician assessment from the clinical photography showed good efficacy and visible aesthetic results for body and face areas. No serious or unexpected adverse side effects were recorded, and transient pain, oedema, erythema and slight induration resolved within the first week of treatment.

**Conclusions:**

The 1444 nm Nd:YAG laser is a new tool for performing lipolysis, and this study reports its effectiveness and safety.

## 1 INTRODUCTION

Body contouring is a significant area of dermatologic and plastic surgery. Surgical procedures, like surgical lipectomy, and less invasive procedures, such as various liposuction techniques, are the two main ways to reduce fat. Different techniques can be used to perform liposuction. There are several common procedures, including suction‐assisted liposuction, ultrasound‐assisted liposuction, power‐assisted liposuction, and laser‐assisted liposuction.^1^ Many medical professionals have experimented with laser lipolysis on different parts of the body. For the purpose of reducing submental fat, Kim and Geronemus^2^ and Goldman^3^ used lipolytic lasers. Laser lipolysis was also used by Prado et al.^4^ to reduce abdominal fat.

Each laser selects a specific chromophore as its target with a different level of affinity. It is clear that the laser's photoacoustic and photothermal effects on fat and water result in lipolysis.^5^, ^6^ According to Anderson et al,^7^ some other wavelengths of laser have a greater affinity for fat than the 1064 nm laser. It has been hypothesized that laser lipolysis can be carried out more successfully with the 1444 nm wavelength since its affinity for fat is more than ten times greater than that of the 1064 nm wavelength.

Additionally, 14% of fat and about 60% of collagen are made up of water.^6^ When compared to the 1064 nm laser, the 1444 nm laser's increased affinity with water was significantly greater than its increased affinity for fat^8^ Consequently, dermal tightening and less bleeding are some of the positive effects of laser lipolysis.

Note that, 1444 nm laser lipolysis is known to be a relatively safe treatment with fewer associated risks and side effects than surgical liposuction for clinical use.^9^ ^–‐^ ^11^

Furthermore, an article recently published^12^ using the 1444 nm Nd:YAG laser in the management of lipoma was found to be a minimally invasive, scar‐free, safe and effective procedure.

Notwithstanding, significant treatment‐related issues, such as permanent scarring, developing large fibrotic areas in the subcutis that lead to irregularity of the overlying skin texture, formation of palpable or visible nodules in the skin, and other possible sequelae could persist during long‐term follow‐up in direct relation to the amount of laser energy used.

Our study showed that 1444 nm Nd:YAG laser‐assisted lipolysis used with appropriate and specific parameters effectively destroys adipose tissue avoiding these risks and determining a safe clinical application.

## 2 MATERIALS AND METHODS

### 2.1 Study protocol and patient population

A subcutaneous, 1,444 nm Nd:YAG laser (LipoAI, Deka M.e.l.a, Florence, Italy) was used on 132 patients (range, 18−73 years; 109 women and 23 men with Fitzpatrick skin phototypes ranging between II and V) requiring body and face contouring.

Patients who were pregnant or breastfeeding, as well as those with a history of keloid formation, were excluded from the study.

There were 11 sites to be treated on the 132 patients, six on the body (upper arms, 11 females; abdomen, 37 (23 females and 14 males); flanks, 17 (11 females and six males); buttocks, eight females; lateral thighs, 34 females; calves and ankles, four females) and 5 on the face (infraorbital fat, one female; lower cheek, three females; nasolabial fold, three females and one male; jowls, two females; and submental area, 11 (nine females and two males).

A cannula with a 600‐m optical fibre was introduced through a 1 mm incision after making sure that the patient and the entire team had appropriate eye protection. The optical fiber can be equipped with a thermal sensor that monitors and maintains the average subdermal temperature below a defined value, and an embedded accelerometer to prevent overheating thanks to a delivery modulation. To make the fibre slightly longer than the cannula, it was threaded through the proximal end of the clamp. The fibre didn't extend further than 2–3 mm even beyond the cannula's end. The fibre tip had to be outside the cannula when the laser emission was turned on. The handpiece was taken out of the treatment area after the procedure. The therapy beam and the targeting beam were coupled into the optical cable from the laser head in order to view the subcutaneous laser action while it was in use. This trans‐illumination effect decreased the chance of cutaneous burns and perforations while providing the surgeon with precise knowledge of where and at what level the Nd:YAG laser was operating. The more intense the aiming light, the more superficial (subdermal) the laser treatment. The laser parameters were selected based on the zone and extension of the area to be treated. For facial areas, the following parameters were used: power, 5–6 W, pulse rate, 20–30 Hz, and the delivered energy varied from 800 to 1500 J. For body areas, the laser setting was as follows: power, 6–7 W, pulse rate, 30–40 Hz, and the delivered energy varied from 1000 to 3500 J.

Palpation, the creation of the ideal contour and shape, and the removal of cannula resistance were used to determine the clinical endpoint. These factors led to lipolysis, which caused the fat tissue to change into an oily, less dense solution. After the procedure, an oily solution containing fat cell debris and membrane‐dissolved lipid was aspirated from the area using a 2‐mm‐diameter cannula and a negative pressure of 0.5 atm (50 kPa or 350–400 mmHg). The incision site was closed with sutures or covered with sterile strips; at the end of the procedure, it was covered with a smooth, non‐adherent antimicrobial dressing, such as compression bandages, for 1‐week post‐procedure. Following surgery, the patients were checked on a regular basis. The follow‐up period ranged from 1 week to 4 months, with 1–2‐months interim visits.

### 2.2 Assessments

All patients were photographed for documentation. Digital clinical photography was taken under as near identical conditions as possible at baseline (pre‐treatment), and 16 weeks post‐treatment.

The 5‐point Global Aesthetic Improvement Scale (GAIS) (0 point—no change; 1 point – 25% mild improvement; 2 points – 50% moderate improvement; 3 points – 75% good improvement; 4 points – 100% excellent improvement) was recorded immediately after treatment and at their final assessment session (4 months). All subjects gave their informed consent before beginning the surgery. Every process was carried out in an outpatient setting, under aseptic conditions, and subsequently, an adequate subcutaneous local anaesthetic/tumescent solution was injected.

### 2.3 Adverse effects

Adverse effects include bleeding, pain, infection, scarring, skin and fat tissue necrosis, itching, numbness, skin contour irregularities, skin discolouration purpura, asymmetry, surgical shock, pulmonary complications, aesthesia, skin loss, hair loss at the treatment area, seroma, allergic reaction, and anaesthesia‐related complications, were monitored.

## 3 RESULTS

**FIGURE 1:**
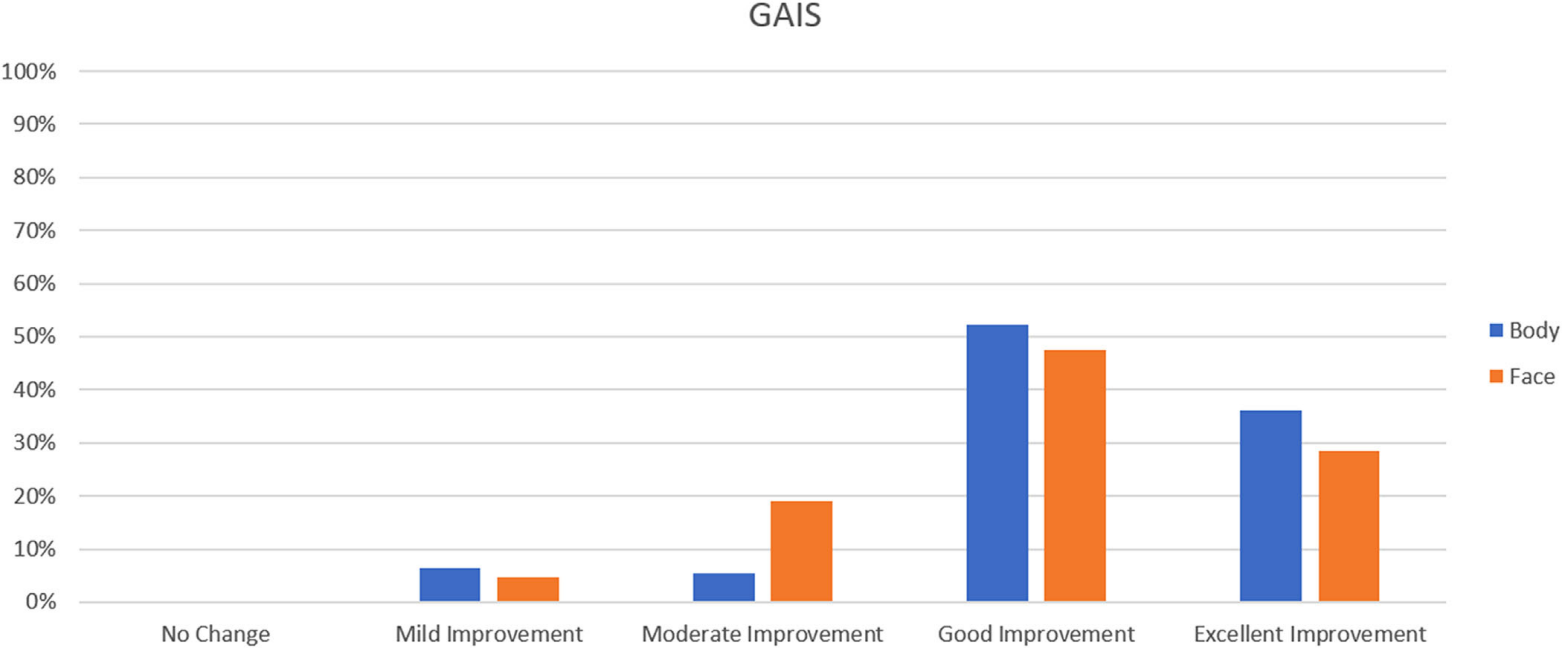
Histogram representing the Global Aesthetic Improvement Scale (GAIS) score results for body and face areas.

**FIGURE 2:**
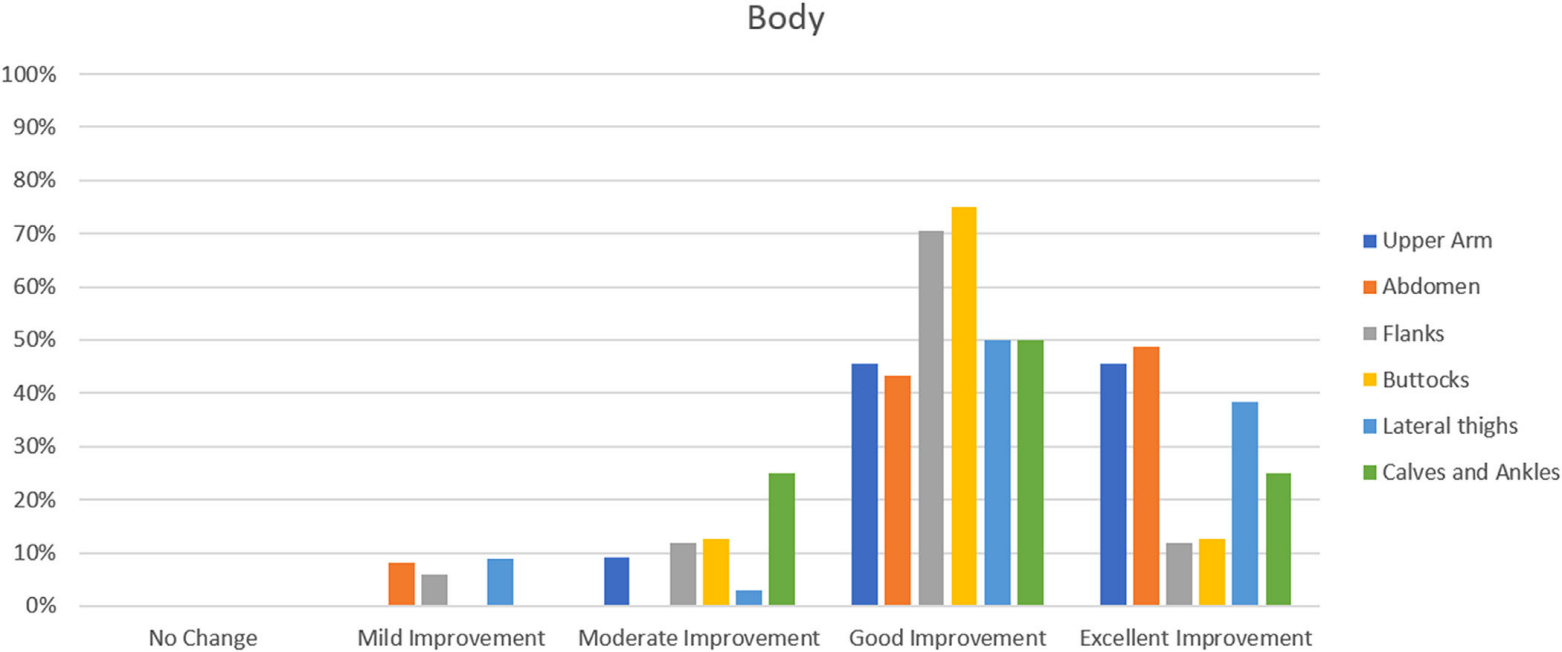
Histogram representing the Global Aesthetic Improvement Scale (GAIS) score results for each body‐treated area.

All patients completed the study. On the basis of the investigator‐evaluated GAIS scale, the total GAIS scores showed satisfactory results: for body areas, most patients, 52%, experienced better than 3‐score (good improvement) changes. Moreover, 36% of the patients experienced better than 4‐score (excellent improvement) changes. None of the patients experienced no change, 6% of patients experienced mild improvement and 5% of patients experienced moderate improvement. For face areas, 0% of patients experienced no change, 5% of patients experienced mild improvement, 19% of patients experienced moderate improvement, 48% experienced good improvement and 29% experienced excellent improvement (Table 1 and Figure 1). In addition, Table 2 illustrates the GAIS score results for each face‐treated area and the GAIS score results for each body‐treated area (see Figure 2) (for upper Arm, 9% of patients experienced moderate improvement, 45% of patients experienced good improvement and 45% of patients experienced excellent improvement; for abdomen, 8% of patients experienced mild improvement, 43% of patients experienced good improvement, 49% of patients experienced excellent improvement; for flanks, 6% of patients experienced mild improvement, 12% of patients experienced moderate improvement, 71% of patients experienced good improvement, 12% of patients experienced excellent improvement; for buttocks, 13% of patients experienced moderate improvement, 75% of patients experienced good improvement, 13% of patients experienced excellent improvement; for lateral thighs, 9% of patients experienced mild improvement, 3% of patients experienced moderate improvement, 50% of patients experienced good improvement and 38% of patients experienced excellent improvement; for calves and ankles, 25% of patients experienced moderate improvement, 50% of patients experienced good improvement, and 25% of patients experienced excellent improvement).

**TABLE 1:** Total Global Aesthetic Improvement Scale (GAIS) score results for body and face areas.

Area	Number of patients	No change	Mild improvement	Moderate improvement	Good improvement	Excellent improvement
Body	111	0%	6%	5%	52%	36%
Face	21	0%	5%	19%	48%	29%

**TABLE 2:** Global Aesthetic Improvement Scale (GAIS) score results for each body and face‐treated area.

Area	Single treated areas	Number of patients	No change	Mild improvement	Moderate improvement	Good improvement	Excellent improvement
Body	Upper Arm	11	0%	0%	9%	45%	45%
	Abdomen	37	0%	8%	0%	43%	49%
	Flanks	17	0%	6%	12%	71%	12%
	Buttocks	8	0%	0%	13%	75%	13%
	Lateral thighs	34	0%	9%	3%	50%	38%
	Calves and Ankles	4	0%	0%	25%	50%	25%
Face	Infraorbital	1	0%	0%	0%	0%	100%
	Lower Cheek	3	0%	33%	0%	67%	0%
	Nasolabial fold	4	0%	0%	50%	25%	25%
	Jowls	2	0%	0%	50%	50%	0%
	Submental	11	0%	0%	9%	55%	36%

**FIGURE 3:**
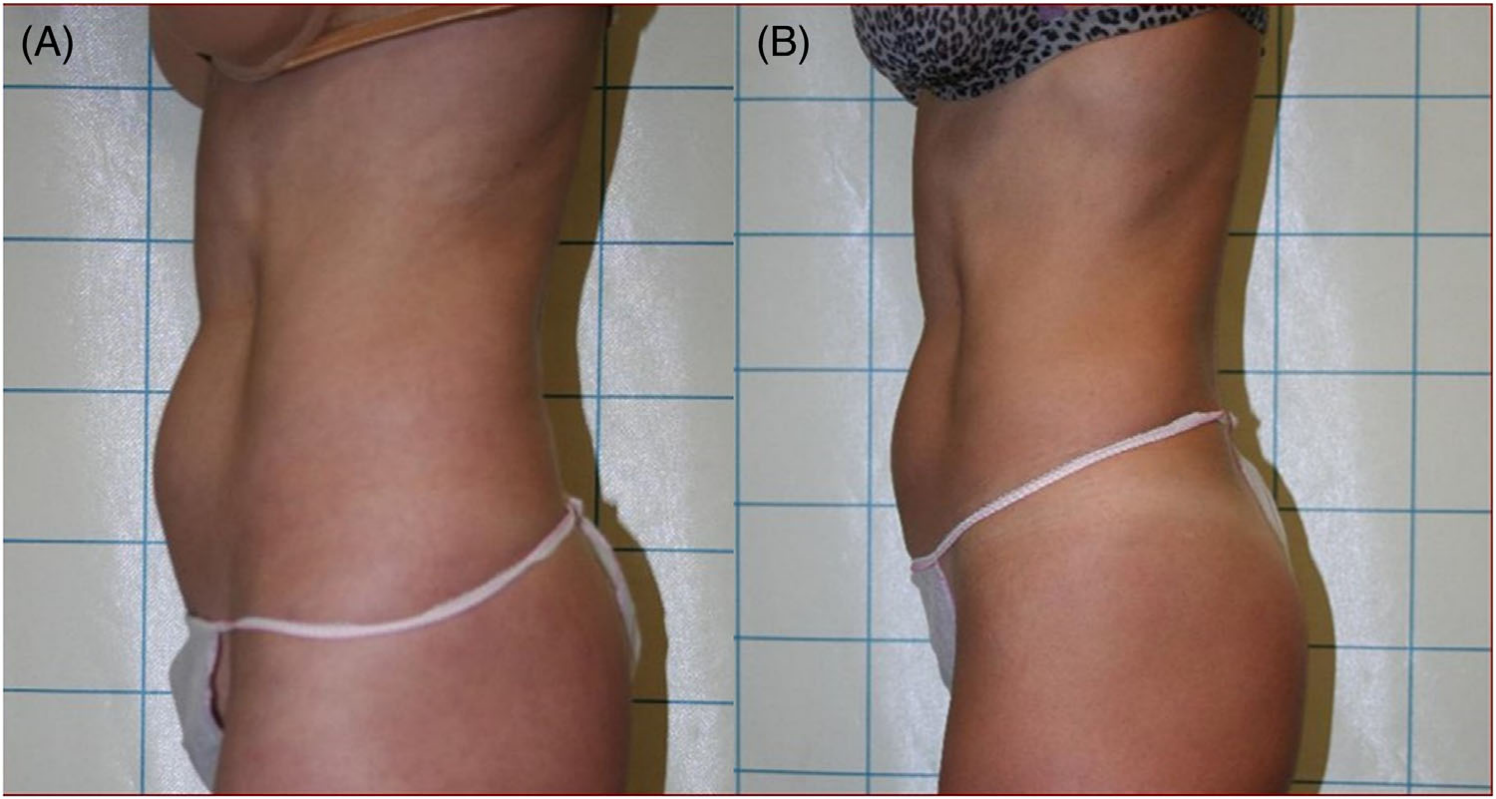
Lateral abdominal view of a female patient before (A) and after (B) 1444 nm Nd:YAG laser lipolysis treatment (4 months follow‐up). Good and visible aesthetic results were achieved.

**FIGURE 4:**
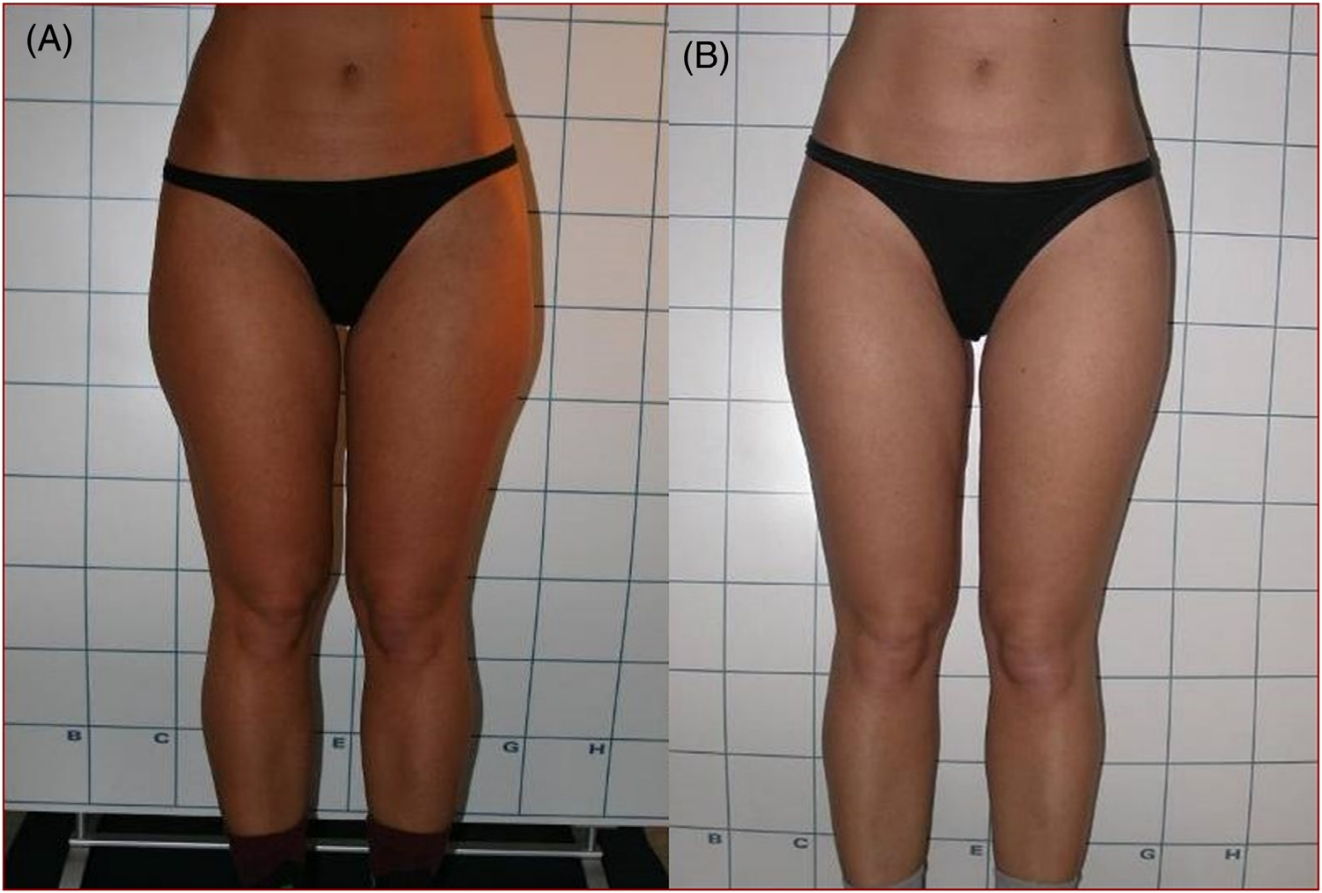
Frontal view of a female patient's flanks before (A) and after (B) 1444 nm Nd:YAG laser lipolysis treatment (4 months follow‐up). The aesthetic outcomes were good and noticeable.

**FIGURE 5:**
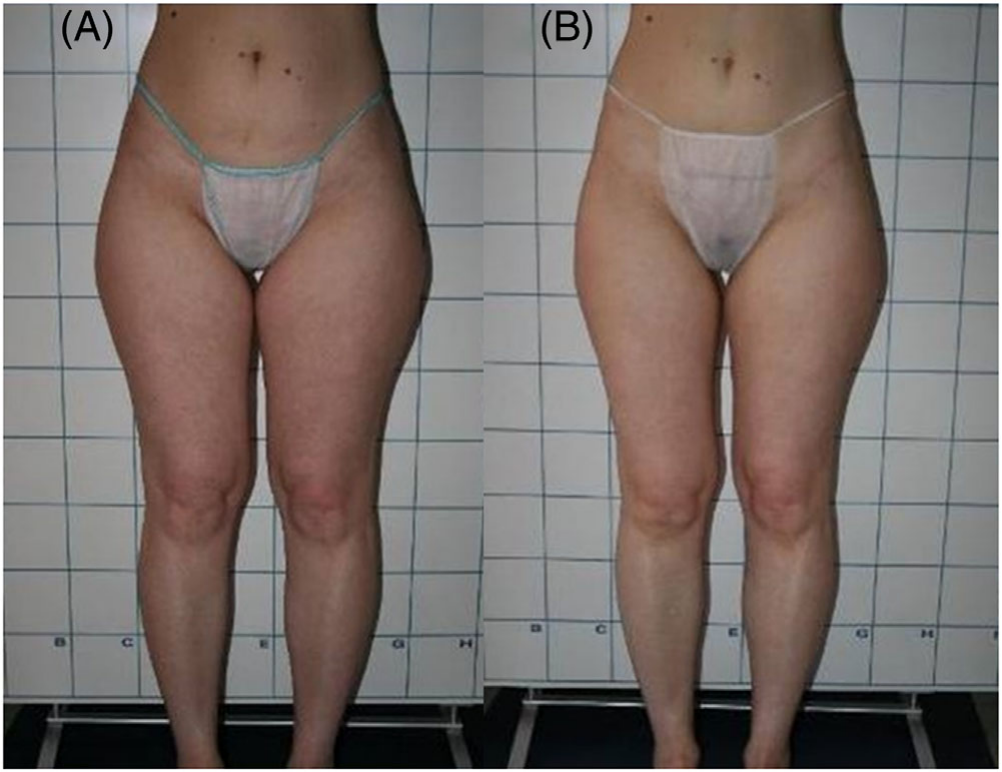
Frontal view of a female patient's flanks before (A) and after (B) 1444 nm Nd:YAG laser lipolysis treatment (4 months follow‐up). The aesthetic outcomes were good and noticeable.

**FIGURE 6:**
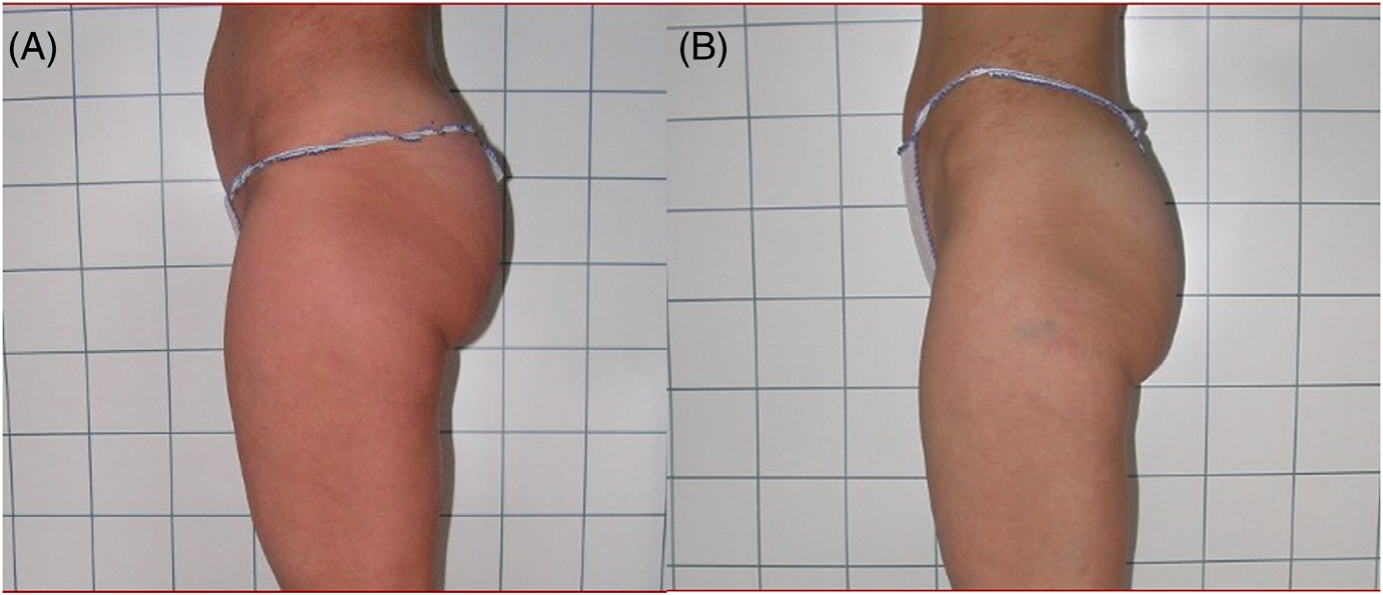
Lateral views of abdomen and culotte of a female patient before (A) and after (B) 1444 nm Nd:YAG laser lipolysis treatment (4 months follow‐up). The aesthetic outcomes were good and noticeable.

Clinician assessment from the clinical photography showed good efficacy and visible aesthetic results for body and face areas. Some of the clinical cases with good aesthetic results are represented in Figures 3, 4, 5, 6. No serious or unexpected adverse side effects were recorded and transient pain, oedema, erythema and slight induration resolved within the first week of treatment.

## 4 DISCUSSION

Scientific investigations have shown that near‐infrared laser‐assisted lipolysis is a fast, secure, and efficient method of removing extra body fat from a variety of locations. However, removing fat from the face has proven to be more challenging.

Histologic studies showed that 1444 nm Nd:YAG laser‐assisted had an efficient lipolytic effect with less inflammation, lipolysis effectively destroys adipose tissue.^11^

The usual method for performing laser lipolysis is to use a cannula with a laser fibre. By inserting the cannula into the treatment area and moving it back and forth, laser energy removes unwanted fat. Dissolved fat can move into the extracellular space thanks to the transient pores that laser‐assisted lipolysis causes in the cell membranes of adipocytes. The laser's short pulse, which is well below the fat's thermal coefficient of relaxation, prevents secondary thermal damage even though the laser energy is instantly converted to heat in that small volume of tissue. With the appropriate and consistent movement of the cannula or fibre, secondary conducted heat is reduced, creating a purely radiant heat effect and nonselective photothermolysis that helps prevent skin overheating and potential burn damage. This is crucial for laser‐assisted facial contouring, as the fat layer and skin are much thinner than in other anatomical parts of the body.

Laser lipolysis offers several advantages: less bleeding, less pain and swelling, dermal tightening, no need for an incision and repair, minimal tissue damage, and early recovery compared to other liposuction methods.^5^, ^13^, ^14^

Our findings confirm these positive lipolytic effects of 1444 nm laser for several different body and face areas. According to photographic documentation and the GAIS scale, satisfactory aesthetic results were achieved following this kind of laser treatment modality.

## 5 STUDY LIMITATION

Study limitations are represented by the lack of ultrasounds and/or histological evaluation with a limited patient's sample. Our future goal will be to increase patient's numbers and to perform valid objective as well as subjective assessments for better analysis of laser treatment performance.

## 6 CONCLUSIONS

The 1444 nm Nd:YAG laser is a new tool for performing lipolysis, and this study reports its effectiveness and safety. The encouraging findings of this study demonstrate that it has significant advantages over other techniques due to its effective lipolytic properties.

## CONFLICT OF INTEREST STATEMENT

I.F. is employed at El.En. Group. The other authors declare that the research was conducted in the absence of any commercial or financial relationships that could be construed as a potential conflict of interest.

## FUNDING INFORMATION

This research received no external funding.

## Data Availability

Data that support the study findings are available on request from the corresponding author.

## References

[1] Mann MW , Palm MD , Sengelmann RD . New advances in liposuction technology. Semin Cutan Med Surg. 2008;27:72–82. 18486027 10.1016/j.sder.2008.01.005

[2] Kim KH , Geronemus RG . Laser lipolysis using a novel 1,064 nm Nd:YAG Laser. Dermatol Surg. 2006;32:241–248. 16442045

[3] Goldman A . Submental Nd: Yag laser‐assisted liposuction Lasers. Surg Med. 2006;38:181–184. 10.1002/lsm.20270 16453321

[4] Prado A , Andrades P , Danilla S , Leniz P , Castillo P , Gaete F . A prospective, randomized, double‐blind, controlled clinical trial comparing laser‐assisted lipoplasty with suction assisted lipoplasty. Plast Reconstr Surg. 2006;118:1032–1045. 16980867 10.1097/01.prs.0000232428.37926.48

[5] Khoury JG , Saluja R , Keel D , Detwiler S , Goldman MP . Histologic evaluation of interstitial lipolysis comparing a 1064, 1320 and 2100 nm laser in an ex vivo model Lasers. Surg Med. 2008;40:402–406. 10.1002/lsm.20649 18649385

[6] Parlette EC , Kaminer ME . Laser‐assisted liposuction: Here's the skinny. Semin Cutan Med Surg. 2008;27:259–263. 19150297 10.1016/j.sder.2008.09.002

[7] Anderson RR , Farinelli W , Laubach H , et al. Selective photothermolysis of lipid‐rich tissues: A free electron laser study. Lasers Surg Med. 2006;38:913–919. 17163478 10.1002/lsm.20393

[8] Tark KC , Jung JE , Song SY . Superior Lipolytic Effect of the 1,444 nm Nd:YAG Laser: Comparison With the 1,064 nm Nd:YAG Laser. Lasers Surg Med. 2009;41:721–727. 20014250 10.1002/lsm.20786

[9] Youn J‐I , Holcomb JD . Ablation efficiency and relative thermal confinement measurements using wavelengths 1,064, 1,320, and 1,444 nm for laser‐assisted lipolysis. Lasers Med Sci. 2013;28(2):519‐527. 22534741 10.1007/s10103-012-1100-9 PMC3586094

[10] Jung YC . Preliminary experience in facial and body contouring with 1444 nm micropulsed Nd:YAG laser‐assisted lipolysis: A review of 24 cases. Laser Ther. 2011;20(1):39‐46. 24155512 10.5978/islsm.20.39 PMC3806076

[11] Lim SD , Youn JI , Kim WS , et al. Comprehensive histologic analysis of interstitial lipolysis with the 1444 nm wavelength during a 3‐month follow‐up. Histol Histopathol. 2011;26(11):1375‐1382. 21938674 10.14670/HH-26.1375

[12] Piccolo D , Mutlag MH , Pieri L , et al. Lipoma management with a minimally invasive 1,444 nm Nd:YAG laser technique. Front Med (Lausanne). 2022;9:1011468. 36479096 10.3389/fmed.2022.1011468 PMC9721359

[13] Katz B , McBean J , Cheung JS . The new laser liposuction for men. Dermatol Ther. 2007;20:448–451. 18093018 10.1111/j.1529-8019.2007.00160.x

[14] Badin AZ , Moraes LM , Gondek L , Chiaratti MG , Canta L . Laser lipolysis: Flaccidity under control. Aesthetic Plast Surg. 2002;26:335–339. 12432470 10.1007/s00266-002-1510-3

